# Dr. Charles Alfred Selby Dalgliesh

**Published:** 1910-09-10

**Authors:** 


Dr. Charles Alfred Selby Dalgliesh, chief surgeon of the Sunderland and Durham County Eye Infirmary, died last week. He was a student of Durham and Oxford, graduating in 1890. Two years later he qualified as a Doctor of Medicine at Durham. He acted successively as house-physician of the Royal Infirmary, Newcastle-on-Tyne, junior and senior resident medical officer of the Radcliffe Infirmary, Oxford, senior house-physician of the General Infirmary, Leeds, and house-surgeon of the Ophthalmic Hospital, Oxford.

